# Poly[[diaqua­hexa-μ-cyanido-cerium(III)ferrate(III)] dihydrate]

**DOI:** 10.1107/S1600536812016911

**Published:** 2012-04-25

**Authors:** Deng-Yong Yu, Xiao-Qing Liu, Ai-Hua Yuan

**Affiliations:** a School of Material Science and Engineering, Jiangsu University of Science and Technology, Zhenjiang 212003, People’s Republic of China; b School of Biology and Chemical Engineering, Jiangsu University of Science and Technology, Zhenjiang 212003, People’s Republic of China

## Abstract

In the structure of the title complex, {[CeFe(CN)_6_(H_2_O)_2_]·2H_2_O}_*n*_, the Ce^III^ and Fe^III^ atoms exhibit square anti­prismatic [CeN_6_(H_2_O)_2_] (site symmetry *m*2*m*) and octahedral [FeC_6_] (site symmetry 2/*m*) coordination geometries, respectively. The metal atoms are linked alternately through the cyanide groups, forming a three-dimensional framework in which the {Ce_2_Fe_2_(CN)_4_} puckered square unit is the basic building block. The crystal packing is enforced by O—H⋯O and O—H⋯N hydrogen bonds, including the uncoordinated water molecule which is located on a mirror plane.

## Related literature

For general background to hexa­cyanido­metalate(III)-based lanthanide complexes, see: Andruh *et al.* (2009 ▶). For related structures, see: Gramlich *et al.* (1990 ▶); Petter *et al.* (1989 ▶).
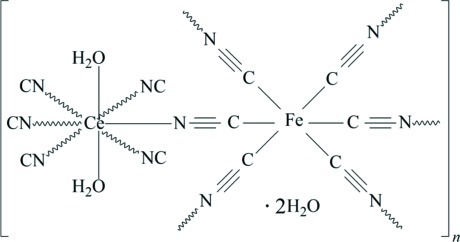


## Experimental

### 

#### Crystal data

[CeFe(CN)_6_(H_2_O)_2_]·2H_2_O
*M* *_r_* = 424.15
Orthorhombic, 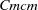

*a* = 7.3806 (11) Å
*b* = 12.7836 (19) Å
*c* = 13.619 (2) Å
*V* = 1285.0 (3) Å^3^
*Z* = 4
Mo *K*α radiation
μ = 4.64 mm^−1^
*T* = 173 K
0.22 × 0.20 × 0.17 mm

#### Data collection

Bruker APEXII diffractometer
Absorption correction: multi-scan (*SADABS*; Bruker, 2004 ▶) *T*_min_ = 0.428, *T*_max_ = 0.506
5578 measured reflections
831 independent reflections
785 reflections with *I* > 2σ(*I*)
*R*_int_ = 0.088

#### Refinement

*R*[*F* ^2^ > 2σ(*F* ^2^)] = 0.037
*wR*(*F* ^2^) = 0.098
*S* = 1.06
831 reflections
51 parameters
H-atom parameters constrained
Δρ_max_ = 1.08 e Å^−3^
Δρ_min_ = −2.69 e Å^−3^

### 

Data collection: *APEX2* (Bruker, 2004 ▶); cell refinement: *SAINT* (Bruker, 2004 ▶); data reduction: *SAINT*; program(s) used to solve structure: *SHELXS97* (Sheldrick, 2008 ▶); program(s) used to refine structure: *SHELXL97* (Sheldrick, 2008 ▶); molecular graphics: *DIAMOND* (Brandenburg, 2006 ▶); software used to prepare material for publication: *SHELXTL* (Sheldrick, 2008 ▶).

## Supplementary Material

Crystal structure: contains datablock(s) I, global. DOI: 10.1107/S1600536812016911/rz2741sup1.cif

Structure factors: contains datablock(s) I. DOI: 10.1107/S1600536812016911/rz2741Isup2.hkl

Additional supplementary materials:  crystallographic information; 3D view; checkCIF report

## Figures and Tables

**Table 1:** Hydrogen-bond geometry (Å, °)

*D*—H⋯*A*	*D*—H	H⋯*A*	*D*⋯*A*	*D*—H⋯*A*
O1—H1*A*⋯O2	0.85	2.08	2.807 (8)	144
O2—H2*B*⋯N1^i^	0.85	2.28	3.126 (11)	177

Symmetry code: (i) 
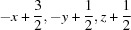
.

**Fig. 1.**
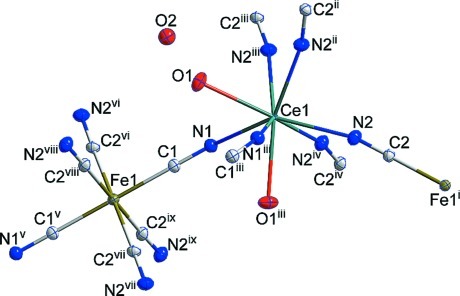
ORTEP diagram of the title complex, showing 30% probability displacement ellipsoidw. Hydrogen atoms have been omitted for clarity. Symmetry codes: (i) -x + 5/2, -y + 1/2, z + 1/2; (ii) -x + 2, y, z; (iii) -x + 2, y, -z + 1/2; (iv) x, y, -z + 1/2; (v) -x + 2, -y, -z; (vi) x - 1/2, -y + 1/2, z - 1/2; (vii) -x + 5/2, y - 1/2, -z + 1/2; (viii) x - 1/2, y - 1/2, -z + 1/2; (ix) -x + 5/2, -y + 1/2, z - 1/2.

**Fig. 2.**
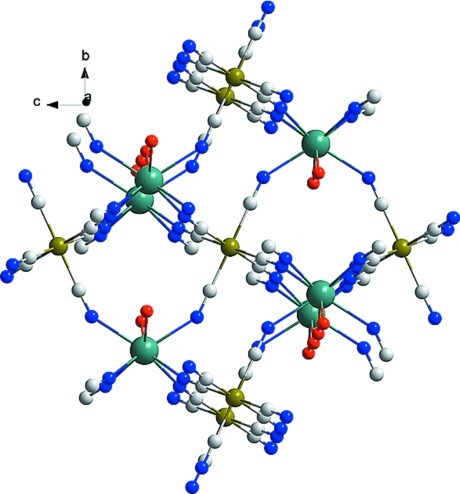
The three-dimensional open framework of the title complex. Hydrogen atoms and uncoordinated water molecules are omitted for clarity.

## supplementary crystallographic information

### Comment

In the past few years, hexacyanometalate-based lanthanide assemblies have received much attention due to their intriguing topologies and interesting functionalities (Andruh *et al.*, 2009). The chelated ligands have played an important role in the construction of low-dimensional complexes. Along this line, we have employed the K_3_Fe(CN)_6_ presusor to react with the Ce^3+^ ion in the presence of the bidentate chelated ligand 3,4,7,8-tetramethyl-1,10-phenanthroline (tmphen). Unexpectly, a new complex Ce(H_2_O)_2_Fe(CN)_6_.2H_2_O was obtained, in which the tmphen ligand was not involved. The structure of the title complex is similiar to those of LnFe(CN)_6_.4H_2_O (Ln = Sm—Lu) reported previously (Gramlich *et al.*, 1990; Petter *et al.*, 1989.).

Single crystal X-ray diffraction analysis revealed that the asymmetric unit of the title complex (Fig. 1) consists of one fourth of a [Ce(H_2_O)_2_]^3+^ cation, one fourth of a [Fe(CN)_6_]^3-^ anion and one half of a water molecules of crystallization. Each iron(III) atom is six-coordinated by six bridging CN groups in a distorted octahedral geometry. The average Fe—C and C—N bond distances are 1.928 (5) and 1.166 (7) Å, respectively. The Fe—CN angles deviate slightly from the linearity, ranging from 178.3 (6) to 178.7 (8)°. Each cerium(III) atom is eight-coordinated with six cyano nitrogen atoms and two oxygen atoms from two coordinated water molecules, forming a square antiprismatic geometry. The Ce—O and the mean Ce—N bond distances are 2.351 (7) and 2.458 (5) Å, respectively. Due to the large ionic radii of the lanthanide atom, the cyanide bridges are exceptionally long and the Ce–N–C bonds are strongly bent with a mean angle of 160.0 (5)°, in opposition to the linearity of the Fe–C—N angle. As a consequence, adjacent Ce and Fe metals are connected through cyano groups to generate a three-dimensional open framework (Fig. 2). The 12-membered puckered square unit Ce_2_Fe_2_(CN)_4_ is the basic building block, in which the Ce and Fe atoms occupy the corners and the CN linkages the edges. The crystal structure is stabilized by O—H···O and O—H···N hydrogen bonds (Table 1).

### Experimental

Single crystals of the title complex were prepared at room temperature by slow diffusion of an ethanol solution (3 ml) of Ce(NO_3_)_3_.6H_2_O (0.10 mmol) and tmphen (0.20 mmol) into an aqueous solution (15 ml) of K_3_[Fe(CN)_6_].H_2_O (0.10 mmol). After about one month, red block crystals were obtained.

### Refinement

All non-hydrogen atoms were refined with anisotropic thermal parameters. The water H atoms were located from a difference Fourier map and refined as riding with O—H = 0.85 Å and *U*(H) set to 1.5*U*_eq_(O).

### Crystal data

**Table:**

[CeFe(CN)_6_(H_2_O)_2_]·2H_2_O	*F*(000) = 808
*M**_r_* = 424.15	*D*_x_ = 2.193 Mg m^−^^3^
Orthorhombic, *C**m**c**m*	Mo *K*α radiation, λ = 0.71073 Å
Hall symbol: -C 2c 2	Cell parameters from 3234 reflections
*a* = 7.3806 (11) Å	θ = 3.0–27.4°
*b* = 12.7836 (19) Å	µ = 4.64 mm^−^^1^
*c* = 13.619 (2) Å	*T* = 173 K
*V* = 1285.0 (3) Å^3^	Block, red
*Z* = 4	0.22 × 0.20 × 0.17 mm

### Data collection

**Table:**

Bruker APEXII diffractometer	831 independent reflections
Radiation source: fine-focus sealed tube	785 reflections with *I* > 2σ(*I*)
Graphite monochromator	*R*_int_ = 0.088
phi and ω scans	θ_max_ = 27.4°, θ_min_ = 3.0°
Absorption correction: multi-scan (*SADABS*; Bruker, 2004)	*h* = −9→9
*T*_min_ = 0.428, *T*_max_ = 0.506	*k* = −16→16
5578 measured reflections	*l* = −17→17

### Refinement

**Table:**

Refinement on *F*^2^	Primary atom site location: structure-invariant direct methods
Least-squares matrix: full	Secondary atom site location: difference Fourier map
*R*[*F*^2^ > 2σ(*F*^2^)] = 0.037	Hydrogen site location: inferred from neighbouring sites
*wR*(*F*^2^) = 0.098	H-atom parameters constrained
*S* = 1.06	*w* = 1/[σ^2^(*F*_o_^2^) + (0.0233*P*)^2^ + 48.4374*P*] where *P* = (*F*_o_^2^ + 2*F*_c_^2^)/3
831 reflections	(Δ/σ)_max_ < 0.001
51 parameters	Δρ_max_ = 1.08 e Å^−^^3^
0 restraints	Δρ_min_ = −2.69 e Å^−^^3^

### Special details

**Table:**

Geometry. All e.s.d.'s (except the e.s.d. in the dihedral angle between two l.s. planes) are estimated using the full covariance matrix. The cell e.s.d.'s are taken into account individually in the estimation of e.s.d.'s in distances, angles and torsion angles; correlations between e.s.d.'s in cell parameters are only used when they are defined by crystal symmetry. An approximate (isotropic) treatment of cell e.s.d.'s is used for estimating e.s.d.'s involving l.s. planes.
Refinement. Refinement of *F*^2^ against ALL reflections. The weighted *R*-factor *wR* and goodness of fit *S* are based on *F*^2^, conventional *R*-factors *R* are based on *F*, with *F* set to zero for negative *F*^2^. The threshold expression of *F*^2^ > σ(*F*^2^) is used only for calculating *R*-factors(gt) *etc*. and is not relevant to the choice of reflections for refinement. *R*-factors based on *F*^2^ are statistically about twice as large as those based on *F*, and *R*- factors based on ALL data will be even larger.

### Fractional atomic coordinates and isotropic or equivalent isotropic displacement parameters (Å^2^)

**Table:**

	*x*	*y*	*z*	*U*_iso_*/*U*_eq_	
C1	1.0000	0.1368 (7)	0.0590 (7)	0.0204 (18)	
C2	1.3141 (9)	0.4530 (5)	0.4106 (5)	0.0208 (13)	
Ce1	1.0000	0.32343 (4)	0.2500	0.0064 (2)	
Fe1	1.0000	0.0000	0.0000	0.0164 (4)	
N1	1.0000	0.2186 (6)	0.0965 (6)	0.0254 (17)	
N2	1.2003 (9)	0.4229 (4)	0.3582 (5)	0.0285 (13)	
O1	0.7401 (11)	0.2171 (6)	0.2500	0.0347 (17)	
H1A	0.7129	0.1879	0.3042	0.052*	
O2	0.5000	0.1562 (6)	0.3993 (6)	0.0342 (17)	
H2A	0.5000	0.0914	0.4131	0.051*	
H2B	0.5000	0.1922	0.4518	0.051*	

### Atomic displacement parameters (Å^2^)

**Table:**

	*U*^11^	*U*^22^	*U*^33^	*U*^12^	*U*^13^	*U*^23^
C1	0.022 (5)	0.022 (4)	0.017 (4)	0.000	0.000	−0.001 (4)
C2	0.021 (3)	0.021 (3)	0.021 (3)	−0.002 (2)	0.000 (3)	−0.003 (2)
Ce1	0.0053 (3)	0.0080 (3)	0.0058 (3)	0.000	0.000	0.000
Fe1	0.0150 (8)	0.0176 (8)	0.0164 (9)	0.000	0.000	0.0002 (6)
N1	0.025 (4)	0.026 (4)	0.025 (4)	0.000	0.000	−0.002 (3)
N2	0.026 (3)	0.033 (3)	0.026 (3)	−0.005 (2)	−0.001 (3)	−0.002 (2)
O1	0.029 (4)	0.049 (4)	0.026 (4)	−0.015 (4)	0.000	0.000
O2	0.035 (4)	0.036 (4)	0.032 (4)	0.000	0.000	0.002 (3)

### Geometric parameters (Å, º)

**Table:**

C1—N1	1.163 (12)	Ce1—N1	2.483 (8)
C1—Fe1	1.925 (9)	Ce1—N1^iv^	2.483 (8)
C2—N2	1.167 (9)	Fe1—C1^v^	1.925 (9)
C2—Fe1^i^	1.930 (7)	Fe1—C2^vi^	1.930 (7)
Ce1—O1	2.351 (7)	Fe1—C2^vii^	1.930 (7)
Ce1—O1^ii^	2.351 (7)	Fe1—C2^viii^	1.930 (7)
Ce1—N2	2.444 (6)	Fe1—C2^ix^	1.930 (7)
Ce1—N2^iii^	2.444 (6)	O1—H1A	0.8503
Ce1—N2^ii^	2.444 (6)	O2—H2A	0.8500
Ce1—N2^iv^	2.444 (6)	O2—H2B	0.8500
			
N1—C1—Fe1	178.7 (8)	N2—Ce1—N1^iv^	76.9 (2)
N2—C2—Fe1^i^	178.3 (6)	N2^iii^—Ce1—N1^iv^	142.05 (16)
O1—Ce1—O1^ii^	109.4 (4)	N2^ii^—Ce1—N1^iv^	76.9 (2)
O1—Ce1—N2	142.58 (15)	N2^iv^—Ce1—N1^iv^	142.05 (16)
O1^ii^—Ce1—N2	78.9 (2)	N1—Ce1—N1^iv^	114.7 (4)
O1—Ce1—N2^iii^	78.9 (2)	C1^v^—Fe1—C1	180.0 (5)
O1^ii^—Ce1—N2^iii^	142.58 (15)	C1^v^—Fe1—C2^vi^	91.1 (3)
N2—Ce1—N2^iii^	117.3 (3)	C1—Fe1—C2^vi^	88.9 (3)
O1—Ce1—N2^ii^	78.9 (2)	C1^v^—Fe1—C2^vii^	88.9 (3)
O1^ii^—Ce1—N2^ii^	142.58 (15)	C1—Fe1—C2^vii^	91.1 (3)
N2—Ce1—N2^ii^	74.4 (3)	C2^vi^—Fe1—C2^vii^	180.0 (4)
N2^iii^—Ce1—N2^ii^	74.2 (3)	C1^v^—Fe1—C2^viii^	88.9 (3)
O1—Ce1—N2^iv^	142.58 (15)	C1—Fe1—C2^viii^	91.1 (3)
O1^ii^—Ce1—N2^iv^	78.9 (2)	C2^vi^—Fe1—C2^viii^	89.4 (4)
N2—Ce1—N2^iv^	74.2 (3)	C2^vii^—Fe1—C2^viii^	90.6 (4)
N2^iii^—Ce1—N2^iv^	74.4 (3)	C1^v^—Fe1—C2^ix^	91.1 (3)
N2^ii^—Ce1—N2^iv^	117.3 (3)	C1—Fe1—C2^ix^	88.9 (3)
O1—Ce1—N1	71.82 (14)	C2^vi^—Fe1—C2^ix^	90.6 (4)
O1^ii^—Ce1—N1	71.82 (14)	C2^vii^—Fe1—C2^ix^	89.4 (4)
N2—Ce1—N1	142.05 (16)	C2^viii^—Fe1—C2^ix^	180.0 (4)
N2^iii^—Ce1—N1	76.9 (2)	C1—N1—Ce1	148.7 (8)
N2^ii^—Ce1—N1	142.05 (16)	C2—N2—Ce1	167.2 (5)
N2^iv^—Ce1—N1	76.9 (2)	Ce1—O1—H1A	116.5
O1—Ce1—N1^iv^	71.82 (14)	H2A—O2—H2B	110.0
O1^ii^—Ce1—N1^iv^	71.82 (14)		

Symmetry codes: (i) −*x*+5/2, −*y*+1/2, *z*+1/2; (ii) −*x*+2, *y*, *z*; (iii) −*x*+2, *y*, −*z*+1/2; (iv) *x*, *y*, −*z*+1/2; (v) −*x*+2, −*y*, −*z*; (vi) *x*−1/2, −*y*+1/2, *z*−1/2; (vii) −*x*+5/2, *y*−1/2, −*z*+1/2; (viii) *x*−1/2, *y*−1/2, −*z*+1/2; (ix) −*x*+5/2, −*y*+1/2, *z*−1/2.

### Hydrogen-bond geometry (Å, º)

**Table:**

*D*—H···*A*	*D*—H	H···*A*	*D*···*A*	*D*—H···*A*
O1—H1*A*···O2	0.85	2.08	2.807 (8)	144
O2—H2*B*···N1^x^	0.85	2.28	3.126 (11)	177

Symmetry code: (x) −*x*+3/2, −*y*+1/2, *z*+1/2.
